# Supplementary material for: The histone chaperone FACT modulates nucleosome structure by tethering its components
Source: Life Sci Alliance. 2018 Jul 10;1(4):e201800107. doi: 10.26508/lsa.201800107 (PMC6238592; doi:10.26508/lsa.201800107)
Supplement: Supplementary file 1 [file LSA-2018-00107_TableS1.pdf]

Table S1: FACT facilitates nucleosome and hexasome assembly. 15- and 1,500-bp DNA fragments are internal markers. MNase 400 U.

Size (bp)	*207 bp Nucleosome*	*207 bp DNA+histone w/o FACT*	*207 bp DNA+histone w/ FACT*
	Molarity (nmol/l)	Molarity (nmol/l)	Molarity (nmol/l)
15	424.4	424.4	424.4
98	0	16.4	0
106	0	0	11.6
115	0	0	9.9
126	34.7	0	19.5
139	6.9	0	0
1500	2.1	2.1	2.1
